# Revisiting the Lipid–Cancer Axis: PCSK9, ANGPTL3, and CETP as Emerging Biomarkers and Therapeutic Targets in Oncology

**DOI:** 10.3390/biom16060831

**Published:** 2026-06-04

**Authors:** Dimitris C. Kounatidis, Natalia G. Vallianou, Fotis Panagopoulos, Antonios Bampiolakis, Vasileios Stamatopoulos, Maria Dalamaga, Iordanis Mourouzis, Constantinos Pantos

**Affiliations:** 1 Diabetes Center, First Department of Propaedeutic Internal Medicine, Laiko General Hospital, Medical School, National and Kapodistrian University of Athens, 11527 Athens, Greece; 2 Department of Pharmacology, National and Kapodistrian University of Athens, 11527 Athens, Greece; imour@med.uoa.gr (I.M.); cpantos@med.uoa.gr (C.P.); 3 First Department of Internal Medicine, Sismanogleio General Hospital, 15126 Athens, Greece; natalia.vallianou@gmail.com (N.G.V.); fotis_1992@hotmail.com (F.P.); mpamp123@gmail.com (A.B.); 4 Emergency Department, Evangelismos General Hospital, 10676 Athens, Greece; kypseli96@gmail.com; 5 Department of Biological Chemistry, Medical School, National and Kapodistrian University of Athens, 11527 Athens, Greece; madalamaga@med.uoa.gr

**Keywords:** ANGPTL3, cancer, CETP, chemotherapy, diagnosis, immune checkpoint inhibitors, lipid metabolism, PCSK9, tumor microenvironment

## Abstract

Cancer remains a major global health challenge, with persistent limitations in early diagnosis, metastatic disease control, and the achievement of durable therapeutic responses with acceptable toxicity. These challenges highlight the need for more precise biomarkers and more effective therapeutic strategies. Increasing evidence implicates dysregulated lipid metabolism as a central contributor to tumor development and progression. In recent years, proprotein convertase subtilisin/kexin type 9 (PCSK9), angiopoietin-like protein 3 (ANGPTL3), and cholesteryl ester transfer protein (CETP) have gained particular attention due to their roles in cholesterol homeostasis, oncogenic signaling, and immune modulation within the tumor microenvironment (TME). This narrative review evaluates the potential of these lipid-regulatory mediators as diagnostic biomarkers and therapeutic targets in oncology. The majority of available evidence derives from preclinical and epidemiological studies, with PCSK9 representing the most extensively investigated target. Findings are sometimes contradictory and strongly influenced by tumor type, disease stage, and biological context, which currently precludes the clinical applicability of these molecules as reliable biomarkers. Similar limitations apply to their translational potential as actionable therapeutic targets. Nevertheless, emerging preclinical evidence suggests that modulation of these glycoproteins may enhance the efficacy of chemotherapy, targeted therapies, and immunotherapy, including nanomedicine-based approaches. Of note, clinical research investigating the role of PCSK9 inhibition in oncology is currently ongoing, whereas comparable studies focusing on ANGPTL3 and CETP remain scarce. Overall, further mechanistic, translational, and prospective clinical investigations are warranted to elucidate the involvement of these lipid-regulatory proteins in cancer biology and to define their potential integration into future oncologic diagnostic and therapeutic strategies.

## 1. Introduction

Cancer remains a major challenge for public health, ranking as the second leading cause of death globally [1]. In the United States alone, nearly two million new cases and approximately 600,000 cancer-related deaths are projected for 2026 [2]. According to the World Health Organization (WHO), the global burden is largely driven by breast, lung, colorectal, and prostate malignancies, while the incidence of several other cancers, such as skin cancers, continues to rise [3].

Despite notable progress in screening and diagnostic tools, many current approaches remain limited in early-stage disease detection and patient risk stratification. Therapeutic strategies have also evolved substantially, with chemotherapy, targeted therapies and immunotherapies now forming the cornerstone of modern oncology. Nevertheless, their clinical benefit is frequently restricted by treatment-related toxicity and pronounced inter-individual variability in response. These limitations underscore the ongoing need for novel biomarkers and complementary therapeutic targets to improve diagnostic precision and patient outcomes [4,5].

Metabolic reprogramming is pivotal in carcinogenesis, with dysregulated lipid metabolism playing a critical role in multiple aspects of tumor growth and progression. Beyond their structural function in cellular membranes, lipids act as essential energy substrates and key regulators of intracellular signaling. Notably, cancer cells demonstrate increased lipid uptake, enhanced de novo lipogenesis, and dynamic lipid remodeling. These processes support rapid proliferation, enable adaptation to metabolic stress, and promote survival under adverse conditions. Importantly, these metabolic alterations are closely integrated with major oncogenic signaling pathways, including the phosphatidylinositol-3-kinase/protein kinase B (PI3K/Akt), mitogen-activated protein kinase (MAPK), and mechanistic target of rapamycin (mTOR) [6,7]. Such adaptations occur within the tumor microenvironment (TME), a complex and highly heterogeneous network composed of both cellular and non-cellular components. The cellular compartment includes immune and stromal cells, while the non-cellular compartment consists of the extracellular matrix (ECM) and extracellular vesicles, known as exosomes [8,9].

Within this framework, increasing attention has been directed toward the impact of lipid-modifying therapies on cancer. A substantial body of evidence, derived mainly from experimental and epidemiological research, suggests that conventional lipid-lowering agents, particularly statins, may possess anti-cancer properties. More recently, interest has expanded to include other lipid-regulatory mediators, including proprotein convertase subtilisin/kexin type 9 (PCSK9), angiopoietin-like protein 3 (ANGPTL3), and cholesteryl ester transfer protein (CETP). Pharmacological inhibition of these glycoproteins leads to pronounced shifts in lipid profiles, including low-density lipoprotein (LDL) cholesterol, high-density lipoprotein (HDL) cholesterol, and triglycerides (TGs), with effects varying according to the underlying mechanism of action. Importantly, with the exception of CETP inhibitors, these agents have demonstrated clinically meaningful reductions in cardiovascular risk and are currently indicated in high- and very high-risk patients, who do not achieve LDL cholesterol goals despite maximally tolerated statin therapy [10,11].

This narrative review examines the roles of PCSK9, ANGPTL3, and CETP in the lipid–cancer axis. It first outlines key biological mechanisms linking lipid metabolism to tumor development and progression, followed by an overview of the physiological roles of these proteins. It then summarizes preclinical and epidemiological data associating these pathways with major solid tumors. Finally, it evaluates the impact of their pharmacological inhibition on the efficacy of established antineoplastic therapies, including nanomedicine-based approaches, and discusses their potential integration into the diagnostic and therapeutic landscape of oncology.

## 2. Literature Search

For this narrative review, a structured literature search was performed using the PubMed database. The search strategy combined the terms “PCSK9”, “ANGPTL3”, “CETP”, “lipid metabolism”, “cancer”, “chemotherapy”, “immunotherapy”, and ‘’nanotechnology’’. Articles published in English up to April 2026 were considered. Eligible studies included original research articles, review papers, clinical investigations, and epidemiological studies, including Mendelian randomization analyses. Studies were selected based on their relevance to the role of PCSK9, ANGPTL3, and CETP in lipid metabolism and cancer biology, as well as their potential implications for cancer treatment and outcomes. To ensure comprehensive coverage, the reference lists of relevant publications were manually screened to identify additional studies of interest. Given the breadth and rapidly evolving nature of this field, the possibility that some relevant studies were not captured cannot be fully excluded.

## 3. The Lipid–Cancer Axis

Lipid metabolism represents a central determinant of malignant transformation and tumor progression. Across diverse cancer types, coordinated alterations in lipid handling support membrane biogenesis, preserve membrane fluidity, and sustain efficient signal transduction under conditions of increased metabolic demand. In this context, cancer cells may exhibit enhanced cholesterol biosynthesis, increased lipoprotein-mediated uptake, and reprogrammed intracellular trafficking pathways [7,12].

Cholesterol, fatty acids (FAs), and phospholipids influence multiple aspects of tumor cell biology, extending far beyond their structural functions to actively regulate oncogenic signaling pathways. In particular, cholesterol acts as a bidirectional regulator of cellular signaling. Oncogenic pathways promote lipid metabolic reprogramming, and cholesterol availability, in turn, modulates the strength and organization of downstream signaling. Cholesterol contributes to pathway activation both through covalent modification of proteins, most notably within the Hedgehog pathway, and through the formation of cholesterol-enriched membrane microdomains [13,14]. These lipid rafts provide a platform for receptor clustering and spatial coordination of major signaling cascades, including MAPK and PI3K/Akt, thereby facilitating proliferation and metabolic adaptation [12,15]. Moreover, cholesterol availability regulates activation of mammalian target of rapamycin complex 1 (mTORC1) at the lysosomal membrane, thereby directly linking lipid status to cellular growth control. Additional implicated pathways include Janus kinase/signal transducer and activator of transcription (JAK/STAT), and wingless-type MMTV integration site family (Wnt). JAK/STAT promotes tumor cell survival, immune modulation, and invasion, whereas Wnt facilitates metabolic adaptation through enhanced glycolysis, glutamine utilization, and nutrient scavenging [12,16,17].

Fatty acid (FA) metabolism represents another highly adaptable axis of lipid dependency in cancer. Tumor cells frequently upregulate de novo lipogenesis, primarily through fatty acid synthase (FASN), to support membrane biosynthesis and promote metastatic potential via epithelial–mesenchymal transition (EMT). Concurrently, the uptake of exogenous lipids is enhanced via transport systems such as cluster of differentiation 36 (CD36), fatty acid transport proteins (FATPs), and fatty acid-binding proteins (FABPs). Fatty acid oxidation (FAO) further increases metabolic flexibility by supplying ATP and biosynthetic intermediates under nutrient-limited conditions, thereby enabling tumor cells to adapt to fluctuating microenvironmental constraints [18,19,20]. Similar to cholesterol metabolism, these processes are closely integrated with signaling networks in a context-dependent manner. Notably, the PI3K/Akt/mTORC1 singling promotes lipogenesis through activation of sterol regulatory element-binding proteins (SREBPs), while AMP-activated protein kinase (AMPK) counteracts this effect during energetic stress [12]. Consistent with these observations, key enzymes such as FASN and stearoyl-CoA desaturase 1 (SCD1) have emerged as central regulators of lipid remodeling across multiple cancer types [21].

Phospholipids complete this functional triad by maintaining membrane architecture, curvature, and cellular motility. Moreover, they serve as precursors for bioactive lipid mediators, particularly sphingolipids such as ceramides and sphingosine-1-phosphate (S1P), which exert context-dependent effects on apoptosis, proliferation, and therapeutic response. Of note, altered phospholipid distribution at the cell surface has been linked to the activation of coagulation pathways, contributing to a pro-tumorigenic systemic environment [22,23].

Lipid metabolic reprogramming extends beyond tumor cells and has been increasingly implicated in shaping the functional landscape of the TME. Dysregulated lipid handling has been associated with altered activity of both adaptive immune cells and myeloid-derived suppressor cells (MDSC). In this context, lipid availability and utilization can differentially affect immune cell subsets, impairing the cytotoxic function of CD8^+^ T lymphocytes while favoring the expansion of regulatory T cells (Tregs). Similarly, lipid metabolism has been linked to the functional polarization of tumor-associated macrophages (TAMs), which exhibit marked plasticity and can adopt either pro-tumor or anti-tumor phenotypes depending on microenvironmental cues. Dendritic cells (DCs) and natural killer (NK) cells are also affected by metabolic constraints within the TME, with potential implications for antigen presentation and early anti-tumor responses. Non-immune stromal components, including endothelial cells and cancer-associated fibroblasts (CAFs), participate in metabolically regulated processes such as angiogenesis, cytokine production, and tissue remodeling [24,25,26]. The non-cellular compartment of the TME also contributes to this interplay. The ECM, largely produced by CAFs, undergoes continuous remodeling during tumor progression and has been linked to cellular metabolic states, including lipid metabolism. This relationship appears to be bidirectional, although the underlying mechanisms remain under active investigation [27]. In addition, exosomes facilitate intercellular communication through the transfer of proteins, nucleic acids, and lipids, thereby contributing to signaling modulation, and metabolic adaptation across both local and distant sites [28].

These mechanisms represent only a subset of the rapidly expanding research on lipid metabolic dysregulation in cancer. Importantly, many of these processes remain incompletely understood and exhibit strong context dependency, varying across tumor types and stages of disease progression. Despite this heterogeneity, accumulating experimental and clinical evidence suggests that targeting lipid metabolic pathways may influence cancer-related outcomes. To date, most studies have focused on traditional lipid-lowering therapies, particularly statins and, to a lesser extent, ezetimibe [10]. More recently, increasing attention has turned to PCSK9, ANGPTL3, and CETP as key molecular nodes linking systemic lipid homeostasis with tumor development and progression. The following sections summarize the roles of these glycoproteins in lipid metabolism and cardiovascular risk reduction, and subsequently examine their potential involvement in tumorigenesis, both as diagnostic biomarkers and as therapeutic targets.

## 4. The Roles of PCSK9, ANGPTL3, and CETP in Lipid Metabolism and Cardiovascular Risk Reduction

### 4.1. PCSK9

PCSK9 is a glycoprotein predominantly produced in the liver, with lower levels of expression in the central nervous system (CNS), lungs, gastrointestinal tract, and kidneys. Its expression is tightly regulated by several transcription factors, including SREBP2 and hepatocyte nuclear factor-1α (HNF-1α). Functionally, PCSK9 plays a central role in cholesterol homeostasis by modulating the availability of low-density lipoprotein receptors (LDLRs) on hepatocytes. These transmembrane receptors bind circulating LDL cholesterol and mediate its hepatic uptake. Under physiological conditions, internalized LDLRs are recycled back to the cell surface. PCSK9 disrupts this process by binding to the LDLR and directing it toward lysosomal degradation, thereby reducing receptor recycling and increasing circulating LDL cholesterol levels [29,30].

Therapeutic targeting of PCSK9 is achieved through two main approaches, with comparable safety and efficacy. Monoclonal antibodies act extracellularly by binding circulating PCSK9 and preventing its interaction with LDLRs, thereby preserving receptor recycling. This class primarily comprises alirocumab and evolocumab, with tafolecimab more recently receiving regulatory approval in China [31,32]. In contrast, small interfering RNA (siRNA)-based agents act intracellularly by suppressing hepatic PCSK9 synthesis through targeted mRNA degradation, with inclisiran currently representing the only available drug. PCSK9 inhibitors are integral to lipid-lowering strategies, particularly in patients at high or very high cardiovascular risk who do not achieve lipid targets despite maximally tolerated statin and ezetimibe therapy. Administered subcutaneously, these medications decrease LDL cholesterol levels by up to approximately 60%, along with modest improvements in TGs and HDL cholesterol [31,33]. Recent data further support the potential of oral PCSK9 inhibition, as enlicitide (20 mg once daily) was associated with a 57.1% reduction in LDL cholesterol levels after 24 weeks of treatment [34].

### 4.2. ANGPTL3

ANGPTL3 is a hepatocyte-derived glycoprotein and a member of the angiopoietin-like protein family. Its C-terminal fibrinogen-like domain (FLD) mediates angiogenic signaling through interaction with integrin αvβ3, thereby influencing endothelial cell adhesion, migration, and survival [35]. In addition, ANGPTL3 inhibits lipoprotein lipase (LPL) and endothelial lipase (EL), underscoring its central role in the regulation of plasma lipid metabolism. Human genetic studies have reported that carriers of ANGPTL3 loss-of-function variants exhibit markedly reduced LDL cholesterol and TG levels, accompanied by a lower risk of cardiovascular disease. Comparable outcomes have also been observed with pharmacological inhibition [36].

Evinacumab, a monoclonal antibody targeting ANGPTL3, is currently the principal therapeutic agent in this class and acts through direct neutralization of the circulating protein. In patients with refractory homozygous familial hypercholesterolemia (HoFH), evinacumab lowers LDL cholesterol levels by approximately 50% and TG levels by 27% [37,38]. Emerging therapeutic approaches include antisense oligonucleotides (ASOs), particularly vupanorsen, as well as siRNA-based therapies such as zodasiran and solbinsiran. These agents primarily target TG metabolism, achieving reductions of up to 60%, along with more modest decreases in LDL cholesterol (~20%), making them especially relevant for the treatment of mixed dyslipidemia [39]. ANGPTL3-targeting therapies are administered by injection and are generally well tolerated. However, vupanorsen has been associated with dose-dependent increases in hepatic fat content, reaching up to 76% in the TRANSLATE-TIMI 70 trial [40].

### 4.3. CETP

CETP is a glycoprotein primarily synthesized in the liver and a member of the lipid transfer protein/lipopolysaccharide-binding protein (LTP/LBP) family. It facilitates the transfer of cholesteryl esters (CEs) from HDL to apolipoprotein B (ApoB)-containing lipoproteins, including LDL, very low-density lipoprotein (VLDL), and chylomicrons, in exchange for TGs transferred from VLDL and chylomicrons to HDL and LDL. Through this bidirectional exchange, CETP plays a key role in reverse cholesterol transport [41].

Pharmacological inhibition of CETP has been investigated with several oral agents, including anacetrapib, dalcetrapib, evacetrapib, and torcetrapib. Although these compounds significantly increased HDL cholesterol and apolipoprotein A1 (ApoA1) levels, they failed to demonstrate meaningful cardiovascular benefits, while some were also associated with adverse safety outcomes [42]. These findings prompted a reassessment of CETP as a therapeutic target and redirected attention toward its potential involvement in other pathological conditions, such as sepsis and age-related macular degeneration. Although the current evidence is promising, it remains preliminary and warrants further investigation [43]. More recently, obicetrapib has renewed interest in this pathway, with effects extending beyond HDL cholesterol elevation alone to reductions in LDL cholesterol, ApoB, and lipoprotein(a) [Lp(a)]. Whether these changes will translate into cardiovascular benefits remains to be established [44,45].

**Figure 1:**
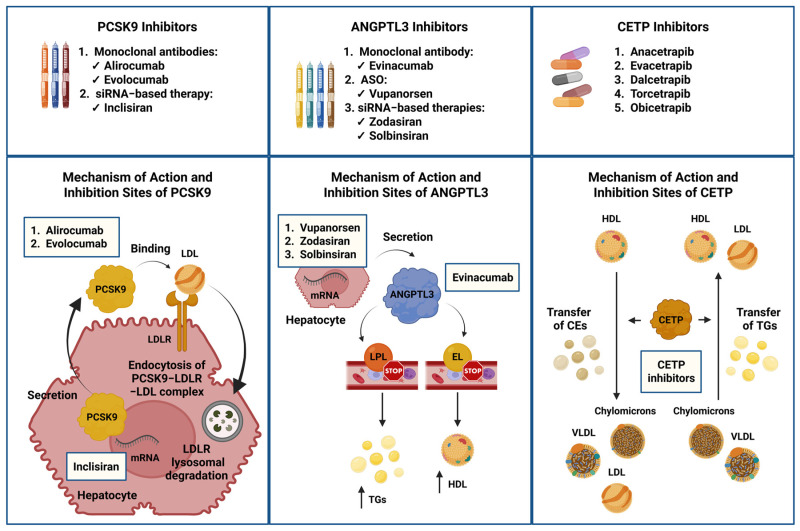
Schematic illustration of the roles of PCSK9, ANGPTL3, and CETP in lipid metabolism, together with the mechanisms of action and sites of activity of their principal pharmacological inhibitors. PCSK9 promotes lysosomal degradation of LDLR, ANGPTL3 inhibits LPL and EL, and CETP mediates the transfer of CEs and TGs between lipoproteins. Therapeutic targeting of PCSK9 and ANGPTL3 is primarily achieved through parenteral approaches, including monoclonal antibodies and siRNA-based therapies, with additional modulation of ANGPTL3 via ASOs. In contrast, CETP inhibition is mediated by orally administered small-molecule agents that directly target the protein [29,31,33,36,37,39,41,42,44]. Abbreviations: ANGPTL3: angiopoietin-like protein 3; ASO: antisense oligonucleotide; CEs: cholesteryl esters; CETP: cholesteryl ester transfer protein; EL: endothelial lipase; HDL: high-density lipoprotein; LDL: low-density lipoprotein; LDLR: low-density lipoprotein receptor; LPL: lipoprotein lipase; PCSK9: proprotein convertase subtilisin/kexin type 9; siRNA: small interfering RNA; TGs: triglycerides; VLDL: very low-density lipoprotein. Created in BioRender. Kounatidis, D. (2026) https://BioRender.com/w0qqlok (accessed on 17 April 2026).

Figure 1 illustrates the mechanisms of action of PCSK9, ANGPTL3, and CETP, as well as the sites at which their principal pharmacological inhibitors exert their effects.

## 5. Brain, Head, and Neck Cancers

### 5.1. Brain Cancers

Brain tumors represent a group of relatively rare malignancies characterized by aberrant and uncontrolled cellular proliferation. In adults, gliomas constitute the most common subtype of primary brain tumors [46]. Recent clinical evidence suggests a potential immunomodulatory role of PCSK9 silencing in glioma. A window-of-opportunity trial evaluating evolocumab in patients with newly diagnosed or recurrent glioma undergoing biopsy or surgical resection showed that the agent was well tolerated and achieved adequate intratumoral concentrations. Notably, higher intratumoral exposure was associated with enhanced antigen presentation, as reflected by upregulation of major histocompatibility complex class I (MHC-I), thereby potentially facilitating CD8^+^ T cell-mediated antitumor immune responses within the TME [47].

In addition, ANGPTL3 has emerged as a candidate prognostic biomarker in glioblastoma. In a cohort of 57 patients, strong ANGPTL3 expression was detected in more than half of tumor samples, while moderate-to-high expression levels were associated with significantly reduced median survival. Multivariate analysis further identified ANGPTL3 expression as an independent predictor of overall survival (*p* = 0.0045). Interestingly, this association was independent of canonical pro-angiogenic signaling pathways, as no correlation was observed with epidermal growth factor receptor (EGFR) or vascular endothelial growth factor receptor (VEGFR) activity [48].

In contrast, direct data linking CETP to brain tumor biology remain absent. However, mechanistic insights into CETP-mediated regulation of CNS lipid homeostasis may provide indirect relevance. A preclinical study by Oestereich et al. reported that increased CETP activity leads to elevated brain cholesterol levels, primarily driven by impaired cholesterol efflux rather than increased de novo synthesis. This dysregulation was accompanied by upregulation of the complement component C1q, which is implicated in cholesterol clearance within the CNS [49]. Within this context, pharmacological CETP inhibition has been proposed as a potential strategy to restore cerebral lipid homeostasis. Supporting its CNS accessibility, evacetrapib has been shown to cross the blood–brain barrier and become detectable in brain tissue within 30 min following intravenous administration (40 mg/kg) [50].

Although these findings are not derived from oncologic models, they suggest a biologically plausible link between CETP-dependent lipid regulation and brain tumor development. Notably, lipid metabolic reprogramming is increasingly recognized as a hallmark of tumor progression, with intracranial tumors demonstrating enrichment in CEs, while activation of the EGFR/PI3K/Akt signaling appears to enhance lipid synthesis and uptake via SREBP-1. Taken together, these observations reinforce the potential relevance of PCSK9, ANGPTL3, and CETP in brain tumor biology, highlighting emerging directions for future research [51].

### 5.2. Head and Neck Cancers

Head and neck cancers (HNC) comprise a group of aggressive malignancies arising from the mucosal epithelium of the oral cavity, pharynx, and larynx, as well as adjacent anatomical structures. The majority of these tumors are histologically classified as head and neck squamous cell carcinomas (HNSCCs), accounting for over 90% of cases. Within this category, oral cancer (OC) and oropharyngeal cancer (OPC) represent two major subtypes with distinct epidemiological and molecular profiles [52].

PCSK9 has emerged as a potential biomarker and therapeutic modulator in HNC, although the current body of evidence is conflicting. For example, Yang et al. showed that PCSK9 is upregulated in HNSCC tissues, with higher expression levels linked to adverse outcomes. In functional models, PCSK9 inhibition attenuated cancer cell stemness in an LDLR-dependent manner, while enhancing CD8^+^ T-cell infiltration and reducing MDSC populations within the TME [53]. In contrast, the work by Kim et al. reported neutral effects of PCSK9 on tumor cell proliferation, migration, and invasion [54]. Moreover, a Mendelian randomization (MR) analysis concluded that genetically proxied PCSK9 inhibition may be correlated with an increased risk of OC and OPC, potentially through mechanisms extending beyond its role in systemic cholesterol regulation [55,56].

Evidence regarding ANGPTL3, although limited, appears comparatively consistent. The study by Koyama et al. revealed significant upregulation of ANGPTL3 in OC tissues (*p* < 0.05), with expression levels positively correlating with tumor size (*p* < 0.05). Furthermore, elevated ANGPTL3 expression in advanced disease was associated with reduced overall survival (*p* < 0.05). Notably, ANGPTL3 silencing suppressed extracellular signal-regulated kinase (ERK) signaling and induced cell-cycle arrest [57]. ANGPTL3 may also contribute to the remodeling of the TME by promoting the differentiation of normal oral fibroblasts into CAFs. This process is accompanied by increased secretion of pro-inflammatory cytokines, including interleukin (IL)-6 and IL-8, as well as upregulation of myofibroblastic markers such as α-smooth muscle actin (α-SMA) and fibroblast activation protein (FAP) [58]. In contrast, the role of CETP in HNC remains largely unexplored. Current genetic evidence provides limited support for a causal association between CETP-mediated LDL cholesterol modulation and the risk of OC or OPC [56].

**Figure 2:**
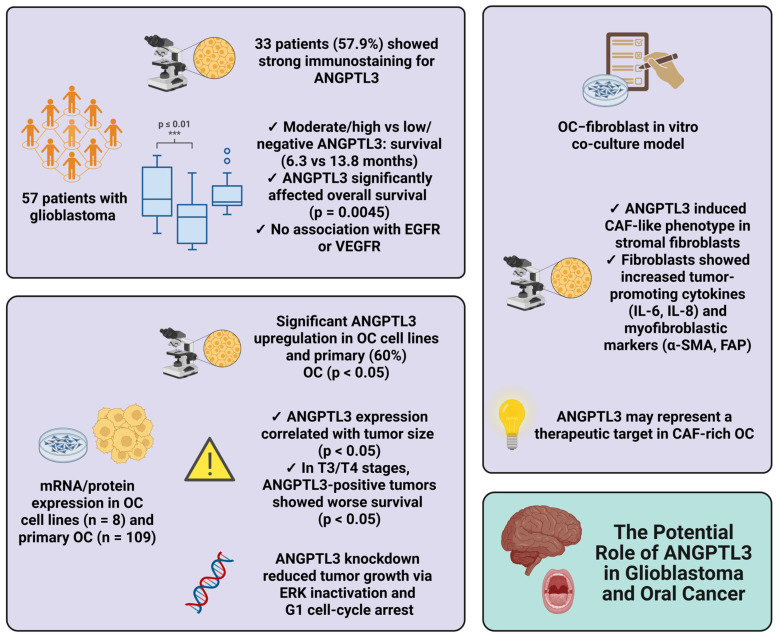
The potential role of ANGPTL3 in glioblastoma and oral cancer. This figure highlights findings from in vitro and clinical research exploring the potential role of ANGPTL3 in tumor development and progression in glioblastoma and oral cancer [48,57,58]. Abbreviations: α-SMA: α-smooth muscle actin; ANGPTL3: angiopoietin-like protein 3; CAF: cancer-associated fibroblast; EGFR: epidermal growth factor receptor; ERK: extracellular signal-regulated kinase; FAP: fibroblast activation protein; IL: interleukin; OC: oral cancer; VEGFR: vascular endothelial growth factor receptor. Created in BioRender. Kounatidis, D. (2026) https://BioRender.com/2r4m8l1 (accessed on 26 April 2026).

Figure 2 depicts findings from in vitro and clinical studies investigating the potential role of ANGPTL3 in tumor development and progression in glioblastoma and OC.

## 6. Lung Cancer

Lung cancer is the leading cause of cancer-related mortality worldwide, largely due to its frequent diagnosis at advanced stages. It is broadly classified into small-cell lung cancer (SCLC) and non-small cell lung cancer (NSCLC). SCLC is characterized by rapid progression and highly aggressive clinical behavior, whereas NSCLC represents the predominant group and includes the main histological subtypes, namely adenocarcinoma, squamous cell carcinoma, and large cell carcinoma. Lipid metabolic processes, including uptake, synthesis, oxidation, and storage, have been shown to contribute to lung cancer development and spread [59,60].

Evidence regarding the involvement of PCSK9 and ANGPTL3 in lung cancer is limited and is largely restricted to NSCLC, while data on CETP are still lacking. Recent findings suggested that PCSK9 may hold prognostic value in NSCLC. In particular, its expression has been proposed as an immunohistochemical marker associated with unfavorable outcomes, especially in patients with resectable disease [61]. Furthermore, preclinical research in lung adenocarcinoma models has shown that siRNA-mediated PCSK9 silencing exerts anti-tumor effects, primarily through induction of mitochondrial dysfunction and activation of endoplasmic reticulum stress (ERS)-related cell death pathways [62]. ANGPTL3 has likewise been implicated in NSCLC, with preliminary data reporting increased expression in tumor tissues. Notably, the combined assessment of circulating ANGPTL3 levels and corresponding autoantibody responses has been suggested to enhance diagnostic accuracy [63]. The pro-tumorigenic effects of ANGPTL3 appear to be partly driven by angiogenesis-related pathways. Cong et al. showed that the long non-coding RNA linc00665 interacts with Y-box binding protein 1 (YB-1), stabilizing it by preventing ubiquitin-mediated degradation and promoting its nuclear translocation. Subsequently, nuclear YB-1 facilitates the transcriptional activation of multiple pro-angiogenic genes, including ANGPTL3, through direct promoter binding [64]. The therapeutic relevance of this pathway is further supported by clinical observations indicating that elevated LPL activity in NSCLC tissue is independently associated with reduced overall survival (*p* = 0.003) [65].

## 7. Gastrointestinal Cancers

### 7.1. Esophageal Cancer

Esophageal cancer is a significant cause of cancer-related mortality, characterized predominantly by two distinct histological subtypes. These include esophageal squamous cell carcinoma (ESCC), which has traditionally accounted for the majority of cases, and esophageal adenocarcinoma (EAC), whose incidence has increased over recent decades. Accumulating evidence indicates that altered lipid handling contributes to tumor progression and aggressiveness and is further implicated in therapeutic resistance and treatment failure [66].

Available evidence suggests a potential role for PCSK9 in esophageal tumorigenesis. Quantitative real-time PCR analyses of paired tumor and adjacent non-tumor tissues from patients with ESCC support the significant upregulation of PCSK9 in malignant epithelium [67]. This overexpression appears to facilitate tumor progression, as PCSK9 has been shown to enhance proliferation, migration, and invasion of ESCC cells. These effects have been linked to the induction of EMT, mediated in part by increased secretion of chemokine (C-C motif) ligand 25 (CCL25) [68]. Host immune responses targeting PCSK9 may also carry prognostic significance, as elevated circulating levels of PCSK9 autoantibodies have been associated with improved postoperative overall survival [69].

ANGPTL3 has also been implicated in esophageal cancer biology. Zhu et al. reported that tumor tissues exhibit significantly higher ANGPTL3 expression compared with adjacent non-neoplastic epithelium (*p* < 0.05). While no consistent correlations with conventional clinicopathological parameters were established, subgroup analyses suggested an age-dependent prognostic role. Specifically, increased ANGPTL3 expression was associated with poorer survival both in younger patients and in those older than 65 years (*p* = 0.021) [70]. Interestingly, ANGPTL3 has been identified as one of nine genes included in a prognostic risk model capable of stratifying patients according to overall survival [71].

### 7.2. Gastric Cancer

In contrast to esophageal cancer, over 90% of gastric tumors are adenocarcinomas. Timely diagnosis is crucial, as it markedly improves patient outcomes. Recent research has highlighted several lipid-related candidate biomarkers, like annexin A11 (ANXA11) and FAP, which may have potential for early detection [72].

High PCSK9 expression has been linked to unfavorable outcomes in gastric cancer. This relationship appears to be driven by enhanced metastatic potential and reduced apoptotic activity, potentially mediated through MAPK signaling. A key component of this mechanism involves the upregulation of heat shock protein 70 (HSP70), a molecular chaperone that stabilizes oncogenic proteins and supports tumor cell survival under stress conditions [73]. Moreover, PCSK9 has been implicated in the regulation of tumor cell proliferation and stemness. Upon activation by TEA domain transcription factor 4 (TEAD4), a downstream effector of the Hippo signaling pathway, PCSK9 promoted these oncogenic properties via modulation of FA metabolism [74].

On the other hand, ANGPTL3 is suggested to exert tumor-suppressive effects in gastric cancer. Lower ANGPTL3 expression has been correlated with poorer overall survival, whereas its experimental overexpression has been shown to inhibit tumor growth and metastatic dissemination [75]. Supporting its potential clinical utility, protein microarray analyses have identified ANGPTL3 as a candidate diagnostic biomarker in gastric cancer [76]. Bioinformatic data have also implicated CETP in gastric cancer progression. Database-driven analyses have identified CETP, along with several additional genes, as a potential biomarker associated with metastatic potential [77]. Furthermore, CETP expression appears to increase with advancing tumor stage in gastric adenocarcinoma. Notably, this stage-dependent pattern may be tumor-specific, as similar associations have not been observed in esophageal cancer within the same datasets [78].

### 7.3. Colorectal Cancer

Colorectal cancer (CRC) is the third most commonly diagnosed malignancy, with a steadily increasing incidence. Prognosis is strongly stage-dependent, with approximately 20–25% of patients presenting with metastatic disease at diagnosis. This substantial disease burden has prompted the implementation of population-based screening programs, typically targeting individuals aged ≥50 years [79]. Evidence indicates that dysregulated lipid metabolism supports the energy demands and biosynthetic requirements of malignant proliferation and facilitates metastatic progression in CRC [80]. Among solid tumors, CRC is one of the most extensively studied in relation to the roles of PCSK9, ANGPTL3, and CETP.

Data support that PCSK9 expression is upregulated in colorectal tumor tissues compared with normal mucosa and exerts predominantly pro-tumorigenic effects. PCSK9 may promote tumor progression and metastatic potential through induction of EMT and activation of the PI3K/Akt signaling. This process is mediated, at least in part, by the upregulation of the transcription factor Snail1, leading to suppression of E-cadherin and concomitant increases in N-cadherin and matrix metalloproteinase-9 (MMP-9) expression. Moreover, experimental data suggest that PCSK9 knockdown influences macrophage polarization through regulation of macrophage migration inhibitory factor (MIF) and lactate metabolism. Given the central role of TAMs in CRC, this shift is functionally significant, as M1-polarized macrophages support anti-tumor immunity, whereas M2 phenotypes promote immune evasion, EMT, and metastatic spread [81,82].

PCSK9 may also intersect with pivotal oncogenic drivers of CRC, particularly Kirsten rat sarcoma viral oncogene (KRAS) mutations. Activation of KRAS sustains MAPK/ERK and PI3K/Akt signaling, while promoting tumor progression, stemness, and remodeling of the TME. Clinically, KRAS-mutant CRC is linked to resistance to anti-EGFR monoclonal antibodies and limited responsiveness to targeted therapies [83]. Notably, PCSK9 overexpression has been shown to enhance de novo cholesterol biosynthesis and increase levels of geranylgeranyl diphosphate, a metabolite required for KRAS activation, thereby correlating with poorer survival in KRAS-mutant CRC. On the other hand, PCSK9 depletion suppressed tumor growth in both in vitro and in vivo models [84].

PCSK9 may further contribute to CRC-associated hepatic metastasis. This effect appears to involve the activation of liver sinusoidal endothelial cells (LSECs), which are key regulators of the hepatic microenvironment. Pre-treatment of LSECs with the PCSK9 inhibitor PF-06446864 reduced the expression of microRNAs associated with migration and proliferation following stimulation by cancer stem cells [85]. Moreover, combined inhibition of PCSK9 and PCSK7 has produced enhanced anti-tumor activity, including reduced liver metastasis and increased CD8^+^ T cell-mediated immune responses [86].

ANGPTL3 has also been involved in CRC progression, particularly in the context of metastatic spread to the liver. Increased ANGPTL3 expression has been associated with tumor progression through MAPK14-dependent signaling [87]. Gene expression analyses further identified ANGPTL3 as one of a limited number of liver-enriched genes that facilitate tumor adaptation to the hepatic microenvironment, thereby promoting metastatic colonization [88]. Research suggests that ANGPTL3 promotes CRC progression and metastasis through interaction with integrin αVβ3, leading to the activation of the focal adhesion kinase (FAK)/janus kinase 2 (JAK2)/STAT3 axis. This cascade drives transcriptional upregulation of COL1A2, contributing to ECM deposition, angiogenesis, and EMT [89].

In contrast to PCSK9 and ANGPTL3, the role of CETP in CRC has been investigated mainly at the clinical and epidemiological level. A case–control study including 99 CRC patients and 101 healthy controls revealed significantly increased CETP activity (*p* < 0.05) and reduced HDL cholesterol levels (*p* < 0.001) in CRC patients. CETP mass emerged as an independent predictor of CRC risk after adjustment for conventional risk factors and oxidative stress markers (*p* < 0.05), suggesting a role for HDL cholesterol metabolism and structural alterations in carcinogenesis [90]. Similar observations have been reported in the European Prospective Investigation into Cancer and Nutrition (EPIC) study, which identified a 23% reduction in colon cancer risk among individuals with higher HDL cholesterol levels, although no significant association was observed for rectal cancer [91]. Collectively, these findings support the hypothesis that CETP inhibition may exert protective effects against CRC, at least in part through HDL-mediated mechanisms [92]. In line with this concept, pharmacological CETP inhibition with evacetrapib has produced direct anti-tumor activity in preclinical CRC models, suppressing cell proliferation via inhibition of Wnt/β-catenin signaling and activation of c-Jun N-terminal kinase (JNK) pathways [93].

**Figure 3:**
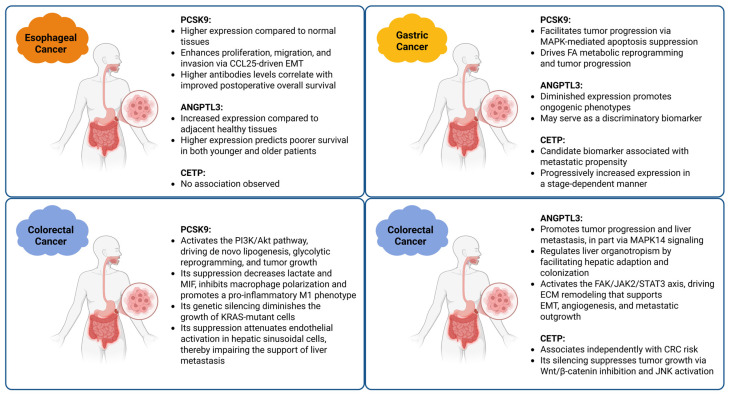
Summary of evidence from preclinical, bioinformatic, and clinical studies on the impact of PCSK9, ANGPTL3, and CETP on major upper and lower gastrointestinal tract malignancies [67,68,69,70,73,74,75,76,77,78,81,84,85,87,88,89,90,93]. Abbreviations: Akt: protein kinase B; ANGPTL3: angiopoietin-like protein 3; CCL25: chemokine (C-C motif) ligand 25; CETP: cholesteryl ester transfer protein; CRC: colorectal cancer; ECM: extracellular matrix; EMT: epithelial–mesenchymal transition; FA: fatty acid; FAK: focal adhesion kinase; JAK2: janus kinase 2; JNK: c-Jun N-terminal kinase; KRAS: Kirsten rat sarcoma viral oncogene; MAPK: mitogen-activated protein kinase; MIF: macrophage migration inhibitory factor; PCSK9: proprotein convertase subtilisin/kexin type 9; PI3K: phosphatidylinositol-3-kinase; STAT3: signal transducer and activator of transcription 3. Created in BioRender. Kounatidis, D. (2026) https://BioRender.com/ete5st2 (accessed on 31 May 2026).

Figure 3 provides a comprehensive overview of the principal mechanisms through which PCSK9, ANGPTL3, and CETP may influence tumorigenesis and progression across major upper and lower gastrointestinal malignancies.

### 7.4. Hepatocellular Carcinoma

Liver cancer is the third leading cause of cancer-related mortality, with hepatocellular carcinoma (HCC) accounting for approximately 90% of primary liver malignancies. Over the past years, its incidence has increased significantly, largely due to the rising prevalence of metabolic dysfunction-associated steatotic liver disease (MASLD). HCC development is characterized by substantial metabolic reprogramming, including enhanced lipid synthesis and uptake, which support tumor growth and progression [94,95]. Given that the liver is the primary site of synthesis for PCSK9, ANGPTL3, and CETP, HCC represents one of the most extensively studied malignancies within this molecular framework.

The role of PCSK9 in HCC is complex and, in several aspects, remains controversial. On one hand, increased PCSK9 expression has been associated with adverse outcomes. Experimental evidence indicated that PCSK9 promotes tumor progression by suppressing apoptosis through upregulation of FASN and inhibition of the Bcl-2-associated X protein (Bax)/B-cell lymphoma 2 (Bcl-2)/caspase-9/caspase-3 signaling cascade [96]. Accordingly, PCSK9 inhibition may influence the p62/Kelch-like ECH-associated protein 1/nuclear factor erythroid 2-related factor 2 (p62/Keap1/Nrf2) antioxidant pathway, thereby inducing ferroptosis and limiting tumor proliferation [97].

Pharmacological inhibition of PCSK9, either alone or combined with simvastatin, may lead to dose-dependent inhibition of proliferation, decreased cell migration, mitochondrial dysfunction, and excessive intracellular lipid accumulation in hepatoma cell lines. Metabolic analyses revealed increased glucose and glutamine consumption and altered mitochondrial respiration, indicating a shift in cellular metabolic programs [98]. Similar effects have been observed with non-pharmacological approaches. For example, arenobufagin, a bioactive component of the traditional Chinese medicine Chan’su, has been shown to mitigate HCC progression by targeting the PCSK9/LDLR pathway to suppress cholesterol synthesis, promote M1 macrophage polarization, induce apoptosis, and inhibit both proliferation and migration of cancer cells [99]. Likewise, far-infrared irradiation enhanced LDLR expression and LDL cholesterol uptake, whereas activation of transient receptor potential vanilloid (TRPV) channels reduced PCSK9 protein levels and exerted anti-tumor effects [100].

In contrast, other findings support a tumor-suppressive role for PCSK9 in HCC. Interaction between PCSK9 and glutathione S-transferase Pi 1 (GSTP1) has been suggested to inhibit JNK signaling, resulting in reduced proliferation and cell cycle progression alongside increased apoptosis in HCC cells [101]. Moreover, PCSK9 overexpression has been associated with attenuation of tumor growth through modulation of the TME, particularly via suppression of M2 macrophage polarization [102]. Notably, near-complete PCSK9 silencing via siRNA markedly increases CD36 expression [103], a key mediator in the transition from MASLD to HCC through enhanced FA uptake within the TME [104].

These apparently contradictory findings suggest that the role of PCSK9 in HCC is highly context-dependent and likely influenced by the metabolic and molecular landscape of the tumor. Important determinants of metabolic dysregulation may include the presence of hyperlipidemia and MASLD, while the TME may differ substantially according to cancer stage. Of note, the tumor-suppressive effects of PCSK9 were not reproduced in hepatoblastoma cell lines, suggesting that its functional impact may additionally depend on the molecular characteristics of individual tumors. Overall, this functional duality complicates the establishment of reliable patient stratification strategies based solely on PCSK9 expression, highlighting the need for integrated molecular, metabolic, and microenvironmental profiling in future studies [96,101,102].

The role of ANGPTL3 in HCC is also unclear. While some data support a pro-tumorigenic function, suggesting that ANGPTL3 may promote tumor growth and invasiveness [105], other evidence suggests a protective role in specific molecular contexts. In these cases, reduced ANGPTL3 expression was associated with improved outcomes, potentially through suppression of zinc finger protein SNAI1 and carnitine palmitoyltransferase 1A (CPT1A), both key regulators of EMT [106]. ANGPTL3 may also contribute to tumorigenesis through metabolic dysregulation in MASLD, acting as a hepatic “fructose sensor” that facilitates fructose uptake and metabolism independently of its LPL inhibitory function [107]. From a translational perspective, elevated ANGPTL3 levels in extracellular vesicles have been proposed as a candidate biomarker for early detection of hepatitis-associated HCC, although its diagnostic utility remains inconsistent across studies [108,109].

Lastly, CETP has emerged as a potential component of multiparametric prognostic models. A urine-based proteomic study in 91 patients developed a non-invasive predictive model for microvascular invasion by integrating CETP with tumor size, serum alpha-fetoprotein (AFP), and gamma-glutamyl transferase (GGT) [110]. Notably, AFP remains an established prognostic marker in HCC, with levels ≥ 400 ng/mL generally associated with poorer survival, although normal AFP levels do not exclude aggressive disease [94]. Beyond individual biomarkers, CETP has also been included in tertiary lymphoid structure (TLS)-related gene signatures, where higher TLS scores correlated with improved survival, distinct immune infiltration patterns, and enhanced predicted response to therapies such as sorafenib, transarterial chemoembolization, and immunotherapy [111]. More recently, a transcriptomic analysis of necroptosis-associated pathways has identified CETP-expressing tissue-resident macrophages with increased susceptibility to necroptosis, supporting a potential role for CETP-positive immune populations as targets for personalized therapeutic strategies [112]. Additional insights are provided by data on apolipoprotein F (ApoF), an endogenous CETP inhibitor, which seems to be upregulated in HCC through promoter activation mediated by ETS proto-oncogenes 1 and 2 and CCAAT/enhancer-binding protein alpha (C/EBPα) [113].

### 7.5. Pancreatic Cancer

Pancreatic cancer is among the most aggressive solid malignancies, with pancreatic ductal adenocarcinoma (PDAC) representing the predominant histological subtype and accounting for approximately 85–90% of cases. Owing to the absence of effective screening strategies, most patients present with advanced or metastatic disease at diagnosis, most commonly involving the liver and lungs. Recent evidence highlights the importance of metabolic reprogramming in PDAC progression. In particular, Yin et al. showed that pancreatic cancer cells undergo extensive lipid remodeling to sustain proliferation and survival under nutrient- and oxygen-deprived conditions. Both lipid anabolic and catabolic processes, including FA and cholesterol metabolism, may drive tumor aggressiveness, representing promising yet complex therapeutic targets [114].

Emerging data support a multifaceted role for PCSK9 in PDAC. Its expression is suggested to be significantly elevated in tumor tissues and has been associated with adverse outcomes, including reduced overall and disease-free survival [115]. Furthermore, circulating PCSK9 levels appear to be increased in individuals with pancreatic cancer compared to those with benign pancreatic disease (BPD), correlating with lymphatic invasion. Notably, PCSK9 may enhance the diagnostic performance of carbohydrate antigen 19-9 (CA19-9), particularly in early-stage disease [116].

These observations are thought to be mediated, at least in part, by the immunomodulatory functions of PCSK9 within the TME. Stromal–tumor crosstalk seems to play a key role in regulating PCSK9 expression. CAFs expressing Lin28b may result in PCSK9 upregulation through activation of the Lin28b–Wnt5a signaling [117]. Increased PCSK9 expression has, in turn, been positively associated with T follicular helper (Tfh) cells and activated DCs, while demonstrating an inverse correlation with monocytes, CD8^+^ T cells, memory B cells, and activated NK cells [115]. Consistent with these findings, PCSK9 has been shown to suppress antitumor immune responses, particularly through inhibition of CD8^+^ T-cell activity. This immunomodulatory capacity has positioned PCSK9 as a promising therapeutic target, especially in the context of T cell-based immunotherapy strategies [118].

Recent data further suggest a role for PCSK9 in shaping metastatic tropism. Tumors with low PCSK9 expression appear to preferentially metastasize to the liver by exploiting lipid-rich hepatic microenvironments and activating mTORC1 signaling. On the other hand, PCSK9-high tumors showed a propensity for lung metastasis, supported by enhanced cholesterol biosynthesis and resistance to ferroptosis [119]. In contrast to PCSK9, evidence regarding ANGPTL3 in pancreatic cancer are lacking, while CETP has been identified as part of TLS-associated gene signatures, suggesting potential prognostic relevance and a possible role in modulating immune organization within the TME [120].

**Figure 4:**
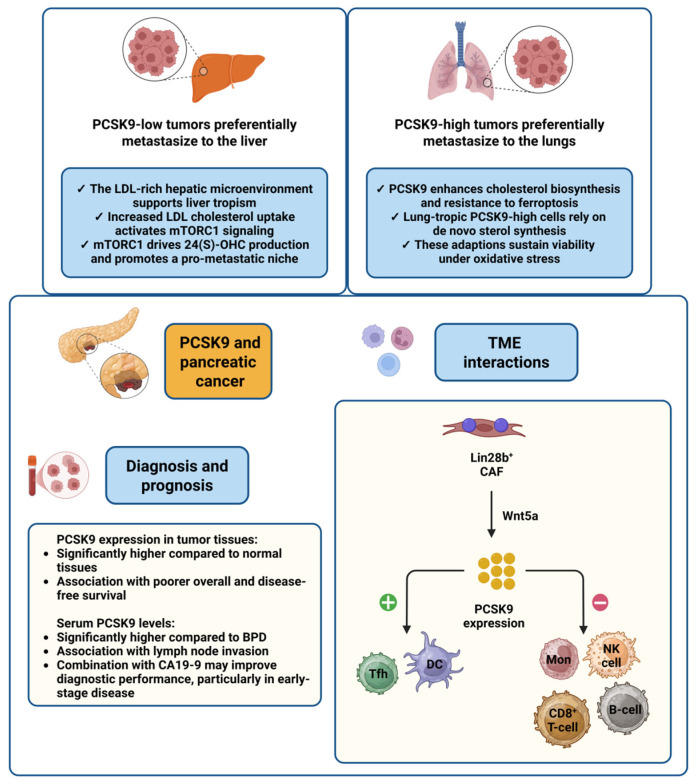
Overview of the current evidence on the role of PCSK9 in pancreatic cancer. This figure compiles available evidence from experimental and clinical studies addressing the role of PCSK9 in pancreatic tumor biology. PCSK9 expression is elevated both in tumor tissues and in the circulation of patients with pancreatic cancer, with reported associations related to both disease diagnosis and prognosis. These associations appear to be mediated, at least in part, through interactions with both immune and stromal components of the TME. Intratumoral PCSK9 expression may play a central role in shaping metastatic potential, particularly with respect to lung and liver dissemination. PCSK9-related lipid metabolism within the TME is a key contributor to this process, differentially modulating mechanisms such as ferroptosis and signaling pathways including mTORC1 [115,116,117,119]. Abbreviations: 24(S)-OHC: 24S-hydroxycholesterol; BPD: benign pancreatic disease; CA19-9: carbohydrate antigen 19-9; CAF: cancer-associated fibroblast; DC: dendritic cell; LDL: low-density lipoprotein; Lin28b: Lin-28 homolog B; mTORC1: mammalian target of rapamycin complex 1; Mon: monocyte; NK: natural killer; PCSK9: proprotein convertase subtilisin/kexin type 9; Tfh: T follicular helper; TME: tumor microenvironment; Wnt5a: wingless-type MMTV integration site family member 5a; +: activate; −: deactivate. Created in BioRender. Kounatidis, D. (2026) https://BioRender.com/4od1zam (accessed on 2 June 2026).

Figure 4 summarizes the current evidence regarding the role of PCSK9 in pancreatic cancer.

## 8. Urological Cancers

### 8.1. Renal Cell Carcinoma

Renal cell carcinoma (RCC) is the predominant primary kidney malignancy. Many cases are detected incidentally during abdominal imaging for unrelated conditions, while management is primarily determined by tumor stage and disease extent. Evidence indicates that renal epithelial cells, particularly under conditions of injury, undergo metabolic reprogramming characterized by increased lipid accumulation, a process that may contribute to tumor initiation and progression [121].

Genetic data suggest a potentially adverse relationship between PCSK9 inhibition and RCC susceptibility, in contrast to ANGPTL3, which exhibited a protective effect. In particular, reduced ANGPTL3 expression has been associated with poorer outcomes, including shorter disease-free and overall survival. At the mechanistic level, ANGPTL3 may limit metastatic capacity by directly binding to vasodilator-stimulated phosphoprotein (VASP) and preventing its phosphorylation at Ser157, thereby disrupting cytoskeletal dynamics and reducing tumor cell motility [122,123]. In addition, ANGPTL3 overexpression may further restrain RCC metastasis through suppression of the Wnt/β-catenin pathway [124].

### 8.2. Bladder Cancer

Bladder cancer (BLCA) is a common and molecularly heterogeneous malignancy characterized by sex-related differences in incidence and outcomes. Prognosis is closely linked to early detection, with urine-based assays used for diagnosis and surveillance. Disease management is stage-dependent, ranging from transurethral resection in non-muscle-invasive cases to radical cystectomy with neoadjuvant chemotherapy in muscle-invasive disease. More recently, immune checkpoint inhibitors (ICIs) have expanded treatment options across disease stages, including advanced and metastatic settings [125].

In BLCA, available evidence is scarce and largely focused on PCSK9. In particular, Wu et al. identified PCSK9 as one of six RNA-binding protein-related factors associated with patient survival, and its inclusion in a multi-gene prognostic model enabled effective risk stratification. Within this signature, elevated PCSK9 expression was linked to poorer overall and recurrence-free survival [126]. Despite these findings, and although dysregulated lipid metabolism has been suggested to play a role in BLCA pathogenesis, recent genetic data do not support a causal relationship between lipid-lowering interventions, including PCSK9 inhibition, and BLCA development [127].

### 8.3. Prostate Cancer

Prostate cancer is the second most commonly diagnosed malignancy in men, with adenocarcinoma being the main histological subtype. Management is risk-adapted, and metastatic disease is primarily treated with androgen deprivation therapy combined with androgen receptor (AR) inhibitors, while chemotherapy is reserved for selected cases. Research has shown that a key feature of malignant transformation is metabolic reprogramming, particularly dysregulated lipid metabolism, which supports the high energetic demands of tumor growth. This involves enhanced lipogenesis driven by FASN, alterations in cholesterol and phospholipid metabolism, and increased FAO mediated by enzymes such as α-methylacyl-CoA racemase (AMACR) [128].

The role of PCSK9 in prostate cancer has been primarily investigated in the context of castration-resistant prostate cancer (CRPC). In particular, PCSK9 inhibition may be associated with a reduced risk of CRPC, a disease state characterized by persistent AR signaling despite androgen deprivation therapy. In this setting, Guo et al. supported that Wnt5a is significantly overexpressed in CRPC tissues, where it increases intracellular free cholesterol levels and activates AR, thereby favoring therapeutic resistance [129]. Inhibition of PCSK9 may also exert beneficial effects by preventing the progression to metastatic castration-resistant prostate cancer (mCRPC). Ahmed et al. showed that the olive leaf polyphenol oleuropein downregulated PCSK9 and restored LDLR function in prostate cancer cells. Through modulation of the PCSK9–LDLR axis, oleuropein supports cholesterol homeostasis and exhibits anti-tumor effects, including reduced proliferation, migration, and recurrence [130].

Recent data extend this lipid-centered framework to ANGPTL3 in metastatic disease. Boulay et al. reported significantly higher circulating ANGPTL3 levels (57.3 ± 26.9 ng/mL, *p* = 0.0390) in patients with metastatic prostate cancer, suggesting potential therapeutic relevance for ANGPTL3 silencing. Notably, within the same cohort, PCSK9 levels were inversely correlated with LDL cholesterol across the study population [131]. To date, no evidence is available regarding the association between CETP and urological cancers.

## 9. Female Cancers

### 9.1. Breast Cancer

Breast cancer is the most frequently diagnosed malignancy in women and remains a leading cause of cancer-related mortality. Early detection is essential, as the disease is often asymptomatic in its initial stages, with population-based mammographic screening substantially improving clinical outcomes. Contemporary management is highly individualized, incorporating chemotherapy, endocrine therapy, anti-human epidermal growth factor receptor 2 (HER2) targeted agents, and immunotherapy according to molecular subtype and tumor stage. Targeting lipid metabolism has emerged as a promising therapeutic strategy in breast cancer, with inhibitors of key lipogenic enzymes such as FASN and SCD1 currently under active investigation [132,133].

An increasing body of research has investigated the role of PCSK9 in breast cancer. PCSK9 has been incorporated into different gene expression-based prognostic models, including a C-X-C motif chemokine ligand 12 (CXCL12)-related signature and an ERS-associated gene panel, both linked to tumor progression and regulation of the immune microenvironment [134,135]. However, its prognostic value is suggested to be subtype-specific, as no clear association has been observed in HER2-positive (HER2^+^) breast cancer despite relatively elevated expression levels [136]. PCSK9 may influence tumor development through modulation of cholesterol metabolism. Silencing of insulin-like growth factor binding protein 6 (IGFBP6) has been shown to reduce LDLR expression while markedly increasing PCSK9 levels, thereby limiting exogenous cholesterol uptake [137]. This regulatory pathway appears particularly relevant in aggressive subtypes such as triple-negative breast cancer (TNBC), where PCSK9-mediated downregulation of LDLR reduces membrane cholesterol content and lipid raft formation. This, in turn, promotes the stimulation of EGFR and HER3 signaling pathways, leading to downstream activation of Src/ERK/c-Jun signaling and increased expression of cyclin D3 and vimentin, ultimately enhancing tumor proliferation and metastatic potential [138]. Despite these mechanistic insights, relevant clinical evidence is limited. A long-term randomized study in premenopausal women at risk for early-stage breast cancer found no significant association between circulating PCSK9 levels and breast cancer outcomes, instead linking PCSK9 more closely to systemic lipid and hormonal profiles [139].

Current data suggest a largely neutral role for ANGPTL3 in breast cancer. Integrated omics analyses have shown that, in contrast to other angiopoietin-like proteins, ANGPTL3 expression is not significantly associated with TME remodeling or patient prognosis [140]. Similarly, clinical data from patients with stage III breast cancer reported no correlation between circulating ANGPTL3 levels and disease status, whereas PCSK9 levels increased with disease severity (*p* < 0.05) [141].

Finally, preclinical research supports a pro-tumorigenic role for CETP in breast cancer. CETP silencing has been shown to reduce cell proliferation by more than 50% in both estrogen receptor-positive (ER^+^) and TNBC cell lines, while in vivo inhibition mitigated tumor growth in xenograft models [142]. These findings are consistent with earlier observations revealing that CETP-deficient cancer cells may exhibit increased susceptibility to intrinsic apoptosis, likely mediated through disruption of cholesterol-dependent pathways, as well as enhanced sensitivity to cytotoxic agents such as tamoxifen [143]. Genetic research also supports a role for CETP in breast cancer risk. MR analyses have linked genetically elevated HDL cholesterol levels to increased breast cancer risk (*p* = 4.9 × 10^−4^), with core HDL pathway loci, including CETP, implicated in this association (*p* = 1.5 × 10^−6^) [144]. In addition, a large-scale analysis involving over 400,000 individuals reported that genetically elevated HDL cholesterol is associated with an increased risk of ER^+^ breast cancer (*p* = 0.037). Notably, variants that raise HDL cholesterol in the gene encoding the target of CETP inhibitors were linked to a higher risk of breast cancer and ER^+^ breast cancer, respectively (*p* = 0.001). The authors concluded that the potential risk-enhancing effects of CETP-mediated increases in HDL cholesterol may have important implications for breast cancer prevention and the design of clinical trials [145]. Of note, CETP has been identified as a susceptibility gene in Afro-American women and has been incorporated into prognostic gene signatures associated with cytochrome c-related pathways [146,147].

### 9.2. Ovarian Cancer

Ovarian cancer is the eighth leading cause of cancer incidence and mortality in women, with approximately 90% being epithelial tumors, most commonly high-grade serous ovarian carcinoma (HGSOC). It is typically diagnosed at an advanced stage due to non-specific symptoms and the absence of effective screening tools. Dysregulated lipid metabolism appears to influence prognosis, as enhanced lipogenesis and lipid uptake may contribute to suppression of antitumor immune responses and chemotherapy resistance [148].

The role of PCSK9 in ovarian cancer remains incompletely defined and somewhat contradictory. Experimental data indicated that PCSK9 overexpression promotes tumor cell survival through activation of key pathways, including Akt and ERK1/2, whereas its inhibition reduces cancer cell viability [149]. In contrast, transcriptomic analyses based on The Cancer Genome Atlas (TCGA) and Gene Expression Omnibus (GEO) datasets suggest that higher PCSK9 expression may be associated with improved overall survival [150].

ANGPTL3 has been more extensively studied in ovarian cancer, with clinical evidence reporting elevated circulating levels in patients with HGSOC [151]. However, findings remain conflicting. On one hand, experimental data support a pro-tumorigenic role. In particular, carcinoma-associated mesenchymal stromal cells (CA-MSCs) enhanced metastatic potential by transferring mitochondria to tumor cells, thereby increasing cellular heterogeneity. This process induced ANGPTL3 secretion, which in turn promoted proliferation of adjacent tumor cells, amplifying tumor progression [152]. These observations are consistent with earlier genomic studies identifying ANGPTL3 within angiogenesis-related gene signatures in HGSOC [153]. On the other hand, higher ANGPTL3 expression has been associated with improved survival, potentially through modulation of the TME. Specifically, ANGPTL3 mitigated metastatic capacity and increased the susceptibility of ovarian cancer cells to NK cell-mediated cytotoxicity and apoptosis. This effect involved IL-2-induced NK cell activation, along with suppression of the JAK2/STAT3 signaling and downstream processes related to immune evasion and ECM remodeling [154].

With respect to CETP, available data are limited but suggest a potential prognostic role. Immunohistochemical analyses have shown significantly increased CETP expression in ovarian cancer tissues compared with normal ovarian samples [155]. Furthermore, CETP has been identified as part of a TLS-related gene signature, alongside C-C chemokine receptor 7 (CCR7) and C-X-C motif chemokine ligand 13 (CXCL13), which is associated with improved survival and enhanced infiltration of CD20^+^ B cells and CD8^+^ T cells, indicating a possible link to anti-tumor immune responses [156].

### 9.3. Cervical Cancer

Cervical cancer is the fourth most common malignancy among women and is primarily driven by persistent infection with high-risk human papillomavirus (HPV). Recent research supports that lipid metabolism plays a role in HPV-related cancer development by supporting viral-driven metabolic reprogramming, which enhances energy production, membrane synthesis, and signaling required for proliferation, invasion, and survival. In addition, lipid alterations contribute to EMT and treatment resistance and may represent potential therapeutic targets in HPV-associated cancers [157].

Available evidence on lipid metabolism-related regulators in cervical cancer is limited. For PCSK9, MR analyses suggested a positive causal association with cervical carcinoma in situ [158]. On the other hand, data regarding ANGPTL3 are derived mainly from experimental studies. ANGPTL3 expression has been reported to be significantly elevated in cervical cancer cells compared with normal cervical tissue, supporting a pro-tumorigenic role. Mechanistically, ANGPTL3 is suggested to promote tumor progression by enhancing angiogenesis via integrin αvβ3-mediated pathways, resulting in increased secretion of vascular endothelial growth factor (VEGF) and its receptor, VEGFR2. Consistently, silencing of ANGPTL3 suppressed cancer cell proliferation, migration, and invasion [159]. Additional indirect evidence further supports this role, as digoxin has been shown to inhibit EMT and metastasis in cervical cancer cells through downregulation of ANGPTL3 via targeting the transcription factor basic helix–loop–helix family member E40 (BHLHE40) [160]. In contrast, no direct data are currently available regarding the role of CETP in cervical cancer. Of note, genetic studies have linked elevated CETP levels with an increased risk of endometrial carcinoma [161].

**Figure 5:**
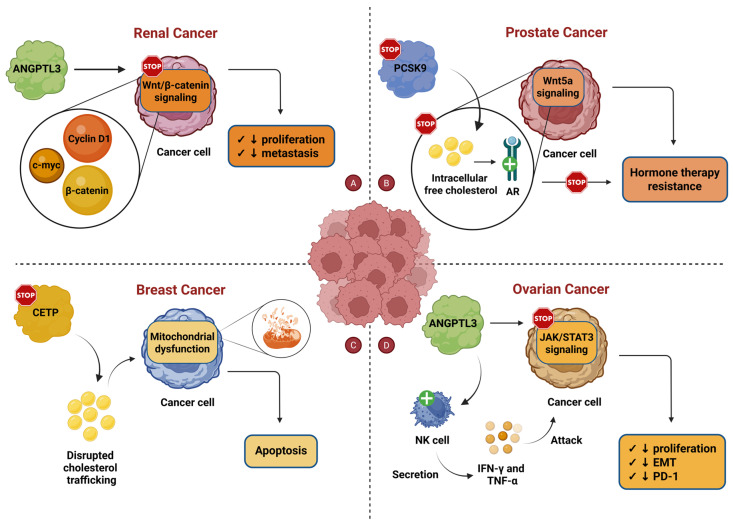
Molecular mechanisms linking PCSK9, ANGPTL3, and CETP to selected urological and female cancers, as reported in experimental studies. (**A**) ANGPTL3 suppresses Wnt/β-catenin signaling, leading to reduced β-catenin activity and downregulation of c-Myc and Cyclin D1, thereby inhibiting tumor proliferation and metastasis [124]. (**B**) Wnt5a promotes intracellular cholesterol accumulation, leading to AR activation and resistance to androgen deprivation therapy in CRPC, while PCSK9 inhibition may counteract this axis [129]. (**C**) CETP inhibition disrupts cholesterol homeostasis, promoting mitochondrial dysfunction and intrinsic apoptosis in breast cancer cells [143]. (**D**) ANGPTL3 inhibits JAK/STAT3 signaling to suppress proliferation, EMT, and PD-1 expression, while enhancing NK cell activation and IFN-γ/TNF-α-mediated cytotoxicity against ovarian cancer cells [154]. Abbreviations: ANGPTL3: angiopoietin-like protein 3; AR: androgen receptor; CETP: cholesteryl ester transfer protein; EMT: epithelial-to-mesenchymal transition; IFN-γ: interferon gamma; JAK: janus kinase; NK: natural killer; PCSK9: proprotein convertase subtilisin/kexin type 9; PD-1: programmed cell death protein 1; STAT3: signal transducer and activator of transcription 3; TNF-α: tumor necrosis factor alpha; +: activate. Created in BioRender. Kounatidis, D. (2026) https://BioRender.com/2rbz2qj. (accessed on 28 May 2026).

Figure 5 illustrates proposed mechanisms by which PCSK9, ANGPTL3, and CETP may influence tumor progression in selected urological and female cancers.

## 10. Cutaneous Melanoma

Cutaneous melanoma represents the most aggressive form of skin cancers, with a steadily increasing incidence, largely attributed to ultraviolet radiation exposure. In line with other solid tumors, dysregulated lipid metabolism may contribute to melanoma progression. Alterations in lipid pathways may also serve as biomarkers of disease behavior, with sphingosine kinase 1 (SphK1) proposed as a marker of progression and resistance to anti-programmed cell death protein 1 (PD-1) therapy [162].

To date, the majority of data regarding lipid-related pathways in melanoma have focused on PCSK9. Experimental evidence suggests that PCSK9 promotes melanoma progression by enhancing tumor cell proliferation, migration, and metastatic dissemination, partly through LDLR-mediated intracellular cholesterol accumulation. The gain-of-function PCSK9 D374Y variant has been associated with increased immune cell infiltration, activation of immune checkpoint pathways, poorer prognosis, and reduced responsiveness to immune checkpoint blockade (ICB). On the other hand, PCSK9 deficiency has been linked to increased apoptotic activity in liver stromal and metastatic cells, accompanied by elevated tumor necrosis factor-α (TNF-α) and reduced Bcl-2 levels [163]. In contrast to these experimental observations, genetic evidence has not demonstrated a clear association between PCSK9 inhibition and melanoma risk. By comparison, genetically proxied inhibition of ANGPTL3 has been correlated with a reduced risk of melanoma, an effect that seems to be independent of its lipid-modifying properties [164].

## 11. Sensitization of Therapeutic Responses and Resistance Modulation

Emerging evidence suggests that modulation of PCSK9 may influence tumor responsiveness to chemotherapy, targeted therapies, and immunotherapy across multiple malignancies. By contrast, data on the therapeutic targeting of ANGPTL3 remain limited, with only minimal data currently available for CETP.

### 11.1. Chemotherapy and Molecular-Targeted Therapies

Contemporary breast cancer treatment incorporates a range of therapeutic agents, including doxorubicin and targeted anti-HER2 therapies, such as trastuzumab. Recent findings suggest that co-administration of evolocumab with these agents in HER2^+^ breast cancer cells may significantly enhance apoptosis and necrosis, partly through modulation of cytokine networks involved in chemoresistance [165]. Additionally, preliminary data support that evolocumab mitigated treatment-related cardiotoxicity via regulation of the myeloid differentiation primary response protein 88/nuclear factor kappa B (MyD88/NF-κB) axis and mTORC1–forkhead box O1/3α (FOXO1/3α) pathways [166]. CETP silencing has also demonstrated therapeutic relevance in breast cancer. Pharmacological blockade of CETP, particularly in combination with cholesterol-depleting agents such as acetyl plumbagin, enhanced tamoxifen-induced apoptosis and improved overall treatment response [142,143].

In HCC, both PCSK9 and ANGPTL3 may play a role in modulating therapeutic response to the tyrosine kinase inhibitor (TKI) sorafenib. Sun et al. reported that PCSK9 inhibition may restore drug sensitivity by suppressing Akt phosphorylation at Ser473, mediated through disruption of PCSK9 palmitoylation at Cys600. This prevented phosphatase and tensin homolog (PTEN) degradation and attenuated pro-survival signaling, thereby counteracting resistance [167]. ANGPTL3 has also been implicated in sorafenib responsiveness. Reduced ANGPTL3 expression characterized resistant HCC cell lines, whereas its re-expression restored sensitivity by suppressing SNAI1 and destabilizing carnitine palmitoyltransferase 1 (CPT1), thereby inhibiting EMT and lipid metabolic reprogramming [106]. In RCC, elevated ANGPTL3 levels have been associated with improved response to sorafenib and enhanced apoptosis, mediated through inhibition of FAK nuclear translocation and reduced p53 ubiquitination [168].

Additional data in ovarian cancer support that PCSK9 expression correlates positively with ANXA11, a protein known to contribute to cisplatin resistance and tumor cell survival [169]. Conversely, ANGPTL3 may enhance sensitivity to paclitaxel through inhibition of the PI3K/Akt/mTOR pathway [170]. Notably, a clinical trial has been designed to evaluate evolocumab in mCRPC, particularly in patients with adverse lipid profiles, to assess its impact on lipid modulation and clinical outcomes [171].

### 11.2. Immunotherapy

The role of lipid metabolism regulators in immunotherapy response has been studied exclusively in relation to PCSK9. PCSK9 silencing has emerged as a promising strategy to enhance the efficacy of ICB, particularly therapies targeting the PD-1/programmed death-ligand 1 (PD-L1) axis [172].

Lao et al. showed that inhibition of SREBP1 attenuated PD-L1 transcription and tumor growth, while PCSK9 regulated PD-L1 protein stability via lysosomal degradation pathways in pancreatic cancer [173]. Additional preclinical studies have also highlighted the potential of PCSK9 inhibition to enhance anti-PD-1 responses across multiple other tumor types, including HNC, NSCLC, HCC, and CRC. A central mechanism underlying this effect is the increased infiltration of CD8^+^ cytotoxic T lymphocytes within the TME, often accompanied by a reduction in Tregs. In HCC, this immunomodulatory effect involves LDLR-dependent activation of mTORC1 signaling, whereas in CRC it may be further potentiated under specific conditions, such as dietary methionine restriction [53,174,175,176,177]. Importantly, these effects may occur independently of systemic lipid lowering. A single mechanistic study supported that, in melanoma and breast cancer cell lines, PCSK9 promotes immune evasion by binding to MHC-I molecules and directing them toward lysosomal degradation. Conversely, PCSK9 inhibition increased tumor-cell surface MHC-I levels, enhanced antigen presentation, and improved CD8^+^ T-cell-mediated antitumor activity [178].

Taken together, these findings support the emerging role of lipid metabolism regulators, particularly PCSK9, as modulators of therapeutic response, with potential implications for overcoming drug resistance and optimizing combination treatment strategies in oncology. Future studies using tumor-specific conditional knockout models or pharmacological approaches selectively targeting PCSK9 within the TME may help distinguish local immunomodulatory effects from systemic lipid-dependent mechanisms.

## 12. Nanotechnology-Enabled Targeting of PCSK9 in Cancer

Nanotechnology-based platforms have emerged as promising tools for targeting PCSK9, enabling synergistic integration with chemotherapy and immunotherapy. In breast cancer models, a nanoliposomal anti-PCSK9 vaccine has elicited robust antibody responses, delayed tumor progression, and improved survival, with enhanced efficacy observed when combined with liposomal doxorubicin [179]. Similarly, virus-like particle-based dual vaccination strategies targeting both HER2 and PCSK9 have improved therapeutic outcomes and delayed resistance to trastuzumab in HER2-positive disease [180].

In CRC, nanotechnology-driven PCSK9 targeting has been successfully combined with immune checkpoint inhibition. A folic acid-targeted DNA tetrahedral nanoparticle co-delivering a PD-L1-binding aptamer and PCSK9-specific siRNA allowed selective tumor accumulation, enhanced T-cell activation, and achieved substantial tumor growth inhibition without systemic toxicity [181]. In addition, in HCC, calcium carbonate (CaCO_3_)-based nanoparticles co-encapsulating doxorubicin and evolocumab have been shown to induce immunogenic cell death, enhance antigen presentation, and potentiate anti-PD-L1 therapy [182]. Recently, advanced nanoCRISPR (Clustered Regularly Interspaced Short Palindromic Repeats) platforms facilitating simultaneous disruption of PD-L1 and PCSK9 have demonstrated the capacity to restore MHC-I-mediated antigen presentation, reverse immune evasion, and sustain tumor control [183].

Collectively, these findings highlight the versatility of nanotechnology in enabling precise modulation of PCSK9 through diverse approaches, including vaccination, RNA interference, and genome editing. In contrast, analogous nanotechnology-based strategies targeting ANGPTL3 or CETP in oncology have not yet been reported. Of note, although similar nanoplatforms have shown promise in other areas of biomedical research, their broader clinical translation remains limited, largely due to challenges such as high development costs and scalability constraints [184].

## 13. Challenges and Future Perspectives

This narrative review provides a comprehensive and updated synthesis of the available evidence regarding the roles of PCSK9, ANGPTL3, and CETP in oncology. These glycoproteins represent relatively novel therapeutic targets in lipid-lowering therapy, with their pharmacological inhibition offering substantial benefits in lipid regulation and, particularly for PCSK9 and ANGPTL3 inhibition, in cardiovascular risk reduction. Given the growing recognition that lipid metabolism may influence tumor biology, an expanding body of research over recent years has suggested that these molecules may also modulate cancer development and progression. In this context, considerable attention has been directed toward investigating their potential both as diagnostic biomarkers and as therapeutic targets in oncology, raising the possibility of repurposing lipid-targeted strategies for oncological applications.

Despite the increasing volume of available data, with PCSK9 dominating the literature, current evidence remains largely preclinical and mechanistic. Moreover, the biological effects of these molecules appear highly context-dependent, with studies reporting divergent and, in some cases, opposing functions across different tumor types and stages of disease progression [96,101,102]. This heterogeneity likely reflects the complex interplay between lipid metabolic reprogramming, oncogenic signaling pathways, and tumor microenvironmental dynamics, underscoring the translational challenges associated with targeting lipid regulatory pathways in cancer [90].

Mechanistic understanding also remains incomplete. Although several studies have explored interactions within the TME, these have predominantly focused on CD8^+^ T lymphocytes, with comparatively limited investigation of stromal components and non-cellular elements. Future research incorporating approaches such as spatial transcriptomics and functional assays may help clarify how these glycoproteins interact with stromal architecture, ECM remodeling, and exosome-mediated signaling within the TME [53,174,175,176,177]. Intriguingly, a recent pan-cancer analysis indicated that PCSK9 dysregulation is frequently driven by epigenetic mechanisms and is associated with distinct patterns of immune infiltration [185].

From a clinical perspective, available data are limited and subject to important methodological constraints. Most studies are based on small cohorts, cross-sectional designs, and lack longitudinal sampling. Variability in assay methodologies and insufficient adjustment for key confounders, including age, metabolic status, and treatment-related effects, further complicate interpretation. Additional limitations include the predominance of single-center cohorts, minimal external validation of findings, and the frequent reliance on narrow or incomplete biomarker panels [90,110,131]. The interpretation of circulating levels is also influenced by systemic factors. For instance, hepatic metastatic involvement may alter measured concentrations due to the liver’s central role in the synthesis of these proteins, while cancer cachexia may disrupt lipid metabolism through increased lipolysis, adipose tissue remodeling, and reduced lipogenesis [90,186]. Future prospective studies should therefore incorporate longitudinal sampling and standardized assay methodologies, along with multivariable adjustment for metabolic, hepatic, and treatment-related confounders. Such approaches may help distinguish cancer-specific biomarker signatures from systemic metabolic fluctuations and improve the diagnostic and prognostic evaluation of the circulating levels of these glycoproteins.

Efforts to infer causality through Mendelian randomization have provided important insights but have yielded inconsistent findings, both in relation to experimental data and across genetic studies. This lack of convergence underscores the complexity of the underlying biology and limits the strength of causal inference. Potential contributors to these discrepancies may include genetic pleiotropy, heterogeneity in the selection and strength of instrumental variables, and differences in study populations and metabolic backgrounds across analyses [56,122,144]. Table 1 summarizes key Mendelian randomization studies evaluating genetically proxied effects of PCSK9, ANGPTL3, and CETP across different cancer types.

These challenges currently preclude the integration of PCSK9, ANGPTL3, and CETP into routine clinical practice as reliable biomarkers or therapeutic targets. Comparable limitations have been observed in other drug repurposing efforts, such as metformin, where, despite extensive research, promising preclinical and observational findings have not translated consistently into clinical benefit. This has largely been attributed to heterogeneity in study populations, and context-dependent effects that are not adequately captured in trial designs [190]. Within this rapidly evolving field, advances in artificial intelligence (AI) may help address these limitations by enabling the identification of clinically relevant patterns in complex, high-dimensional datasets, thereby improving biomarker discovery and therapeutic stratification [191].

Despite these challenges, and although early clinical trials have reported limited efficacy despite favorable safety outcomes (NCT05128539) or have been suspended pending protocol modifications (NCT06385262), ongoing studies are expected to yield important insights into the potential role of PCSK9 silencing in cancer. These trials may help clarify the value of PCSK9 inhibition as an adjunct to standard-of-care treatments and immunotherapy across a broad range of solid tumors. Current investigations include both established agents and next-generation antibodies such as tafolecimab and tolecizumab, with a primary focus on lung cancer and CRC. By contrast, comparable clinical exploration of ANGPTL3 and CETP silencing strategies in oncology is still lacking. Table 2 summarizes ongoing clinical trials exploring PCSK9-targeted strategies in combination treatment settings.

## 14. Conclusions

This work synthesizes available evidence positioning PCSK9, ANGPTL3, and CETP as emerging mediators within the lipid–cancer axis, with potential implications for biomarker development and therapeutic intervention. Despite growing interest, current knowledge remains heavily weighted toward preclinical studies, with limited clinical validation, thereby restricting definitive conclusions regarding causality and clinical applicability. Consequently, the translational maturity of this field remains limited, precluding, at present, the routine clinical implementation of these molecules, particularly in diagnostic settings. Ongoing clinical trials, predominantly focused on PCSK9, are expected to provide critical insights into the feasibility and therapeutic relevance of targeting lipid-regulatory pathways in cancer. In contrast, ANGPTL3- and CETP-directed strategies remain underexplored in the oncologic context, underscoring a clear gap in clinical investigation. Addressing this gap will be essential to delineate the context-specific roles of these molecules across tumor types and disease stages. Ultimately, a more comprehensive understanding of the mechanistic interplay between lipid metabolism and tumor biology will determine whether modulation of these pathways can be effectively translated into improved clinical outcomes and inform their integration into precision oncology frameworks.

## Figures and Tables

**Table 1:** Genetically proxied effects of PCSK9, ANGPTL3 and CETP on cancer risk across Mendelian randomization studies.

Author, Year	Source of Data	Results
Gormley et al., 2021 [56]	GWAS data from the GAME-ON and the UK Biobank, with lipid trait instruments derived from large GWAS meta-analyses	✓ PCSK9 inhibition is associated with increased OC/OPC risk (*p* = 9.31 × 10^−5^), with the association appearing stronger for OPC ✓ CETP shows no significant association
Liu et al., 2021 [122]	GWAS data from the International Agency for Research on Cancer (IARC) and the National Cancer Institute (NCI)	Genetically proxied PCSK9 inhibition is associated with increased risk of RCC in men
Cheng et al., 2023 [127]	GWAS data from the UK Biobank and the FinnGen Project	No causal association is observed between genetically proxied PCSK9 inhibition or evinacumab and bladder cancer risk
Johnson et al., 2020 [144]	GWAS data from the Million Veteran Program (MVP) and the Breast Cancer Association Consortium (BCAC)	✓ Genetically elevated HDL cholesterol is associated with increased breast cancer risk in MR analysis (*p* = 4.9 × 10^−4^) ✓ Core HDL pathway variants, including CETP, are associated with breast cancer risk (*p* = 1.5 × 10^−6^)
Nowak et al., 2018 [145]	Two-sample Mendelian randomization using GWAS data	✓ LDL cholesterol lowering variants mimicking PCSK9 inhibitors are correlated with lower breast cancer risk (*p* = 0.014) ✓ Genetically elevated HDL cholesterol is associated with an increased risk of ER^+^ breast cancer (*p* = 0.037) ✓ HDL cholesterol-raising variants in the gene encoding the target of CETP inhibitors are linked to higher risk of breast cancer and ER^+^ breast cancer (*p* = 0.001)
Wang et al., 2024 [158]	OpenGWAS	✓ Genetically proxied PCSK9 inhibition is associated with: Reduced risk of breast (*p* = 2.25 × 10^−2^) and lung cancer (*p* = 2.55 × 10^−3^) Increased risk of gastric cancer (*p* = 1.88 × 10^−2^), hepatic cancer (*p* = 1.16 × 10^−2^), OC/OPC (*p* = 3.36 × 10^−4^), and cervical carcinoma in situ (*p* = 6.91 × 10^−3^) ✓ Genetically proxied PCSK9 inhibition is not associated with bladder, thyroid, pancreatic, colorectal, kidney, brain, or esophageal cancers
Che et al., 2024 [164]	GWAS data from two independent lipid genome-wide association study datasets	✓ No robust association is observed between PCSK9 inhibition and melanoma risk ✓ ANGPTL3 inhibition is associated with reduced melanoma risk
Fang et al., 2023 [187]	GWAS data from the Global Lipids Genetics Consortium and the PRACTICAL consortium	Genetically proxied PCSK9 inhibition is associated with reduced risk of total prostate cancer (*p* = 9.15 × 10^−3^) and early-onset prostate cancer (*p* = 0.023)
Chen et al., 2023 [188]	GWAS data from the Global Lipids Genetics Consortium	✓ ANGPTL3 inhibition is associated with reduced risk of CRC and gastric cancer (q < 0.05) ✓ No cancer-specific associations were observed for PCSK9 or CETP
Li et al., 2024 [189]	GWAS data from published lipid GWAS studies	No significant association is reported between genetically proxied PCSK9 inhibition and ovarian or cervical cancer risk

Abbreviations: ANGPTL3: angiopoietin-like 3; CETP: cholesteryl ester transfer protein; ER^+^: estrogen receptor-positive; GWAS: genome-wide association study; HDL: high-density lipoprotein; LDL: low-density lipoprotein; MR: Mendelian randomization; OC: oral cancer; OPC; oropharyngeal cancer; PCSK9: proprotein convertase subtilisin/kexin type 9; RCC: renal cell carcinoma.

**Table 2:** Ongoing clinical trials evaluating PCSK9-targeted therapeutic strategies in oncology.

Study ID	Treatment Regimen	Cancer Type	Study’s Objective
NCT07014215	Tafolecimab plus bevacizumab plus sintilimab	Advanced NSCLC	To evaluate efficacy (primary endpoint: PFS) and safety of adding PCSK9 inhibition and anti-angiogenic therapy to PD-1 blockade in patients with acquired resistance to prior PD-1/PD-L1 therapy
NCT05144529	Evolocumab plus nivolumab/ipilimumab	Treatment-naïve metastatic NSCLC	To assess safety, tolerability, and immunological impact of adding evolocumab to standard nivolumab/ipilimumab, and explore potential enhancement of anti-tumor activity
NCT05553834	Alirocumab plus cemiplimab	Metastatic, refractory NSCLC	To investigate whether combining PCSK9 inhibition with PD-1 blockade can overcome resistance and induce anti-tumor activity in patients who progressed after prior PD-1 therapy
NCT07061535	Tafolecimab plus sintilimab plus platinum–etoposide chemotherapy	Extensive-stage SCLC	To evaluate efficacy (primary endpoint: PFS), safety, and biomarker effects of adding PCSK9 inhibition to chemoimmunotherapy, including the impact on MHC-I expression and response prediction
NCT07468630	Tolecizumab plus sintilimab plus CapeOX	Locally advanced colon adenocarcinoma	To assess efficacy and safety of adding PCSK9 inhibition to neoadjuvant chemoimmunotherapy, with primary endpoint pCR and secondary endpoints including MPR, ORR, R0 resection rate, PFS, and OS
NCT06304987	Tafolecimab plus sintilimab plus neoadjuvant chemoradiotherapy	Locally advanced middle/low rectal cancer	To compare efficacy and safety of adding PCSK9 inhibition to PD-1-based neoadjuvant chemoradiotherapy, focusing on complete response rates and survival outcomes
NCT06933251	PCSK9 inhibitor plus PD-1 inhibitor plus neoadjuvant chemoradiotherapy	Locally advanced rectal cancer	To evaluate complete response rates and survival outcomes of dual PCSK9/PD-1 blockade combined with neoadjuvant chemoradiotherapy, and assess safety
NCT06391905	PCSK9 inhibitor plus standard first-line therapy	Advanced CRC	To evaluate efficacy and safety of adding PCSK9 inhibition to first-line treatment, and explore predictive immune biomarkers
NCT04862260	Evolocumab plus atorvastatin plus ezetimibe plus FOLFIRINOX	Locally advanced or metastatic pancreatic adenocarcinoma	To evaluate feasibility and preliminary efficacy of inducing cholesterol depletion alongside standard chemotherapy to inhibit tumor progression and enhance treatment response
NCT06284564	Evolocumab plus nivolumab	Metastatic, refractory RCC	To explore anti-tumor activity (response rate) and safety of evolocumab plus nivolumab in patients refractory to immunotherapy and/or VEGF-targeted therapy

Abbreviations: CapeOX: capecitabine plus oxaliplatin; CRC: colorectal cancer; FOLFIRINOX: 5-Fluorouracil, Leucovorin, Irinotecan, and Oxaliplatin; MHC-I: major histocompatibility complex class I; MPR: major pathological response rate; NSCLC: non-small cell lung cancer; ORR: objective response rate; OS: overall survival; pCR: pathological complete response rate; PCSK9: proprotein convertase subtilisin/kexin type 9; PD-1: programmed cell death protein 1; PD-L1: programmed death-ligand 1; PFS: progression-free survival; RCC: renal cell carcinoma; SCLC: small cell lung cancer; VEGF: vascular endothelial growth factor.

## Data Availability

No new data were created or analyzed in this study.

## Abbreviations

24(S)-OHC: 24S-hydroxycholesterol; α-SMA: α-smooth muscle actin; AFP: alpha-fetoprotein; AI: artificial intelligence; Akt: protein kinase B; AMACR: α-methylacyl-CoA racemase; AMPK: AMP-activated protein kinase; ANGPTL3: angiopoietin-like protein 3; ANXA11: annexin A11; ApoA1: apolipoprotein A1; ApoB: apolipoprotein B; ApoF: apolipoprotein F; AR: androgen receptor; ASO: antisense oligonucleotide; Bax: Bcl-2-associated X protein; Bcl2: B-cell lymphoma 2; BHLHE40: basic helix–loop–helix family member E40; BLCA: bladder cancer; BPD: benign pancreatic disease; CA 19-9: carbohydrate antigen 19-9; CAFs: cancer-associated fibroblasts; CA-MSCs: carcinoma-associated mesenchymal stromal cells; CapeOX: capecitabine plus oxaliplatin; CCL25: chemokine (C-C motif) ligand 25; CCR7: C-C chemokine receptor 7; CD36: cluster of differentiation 36; CEs: cholesteryl esters; CETP: cholesteryl ester transfer protein; CNS: central nervous system; CPT1A: carnitine palmitoyltransferase 1A; CRISPR: Clustered Regularly Interspaced Short Palindromic Repeats; CRC: colorectal cancer; CRPC: castration-resistant prostate cancer; CXCL12: C-X-C motif chemokine ligand 12; CXCL13: C-X-C motif chemokine ligand 13; DCs: dendritic cells; EAC: esophageal adenocarcinoma; ECM: extracellular matrix; EGFR: epidermal growth factor receptor; EL: endothelial lipase; EMT: epithelial–mesenchymal transition; EPIC: European Prospective Investigation into Cancer and Nutrition; ER^+^: estrogen receptor-positive; ERK: extracellular signal-regulated kinase; ERS: endoplasmic reticulum stress; ESCC: esophageal squamous cell carcinoma; ETS: E26 transformation-specific; FAs: fatty acids; FABPs: fatty acid-binding proteins; FAK: focal adhesion kinase; FAP: fibroblast activation protein; FASN: fatty acid synthase; FATPs: fatty acid transport proteins; FLD: fibrinogen-like domain; FOLFIRINOX: 5-Fluorouracil, Leucovorin, Irinotecan, and Oxaliplatin; FOXO1/3α: forkhead box O1/3α; GEO: Gene Expression Omnibus; GSTP1: glutathione S-transferase Pi; GWAS: genome-wide association study; HCC: hepatocellular carcinoma; HDL-C: high-density lipoprotein cholesterol; HER2^+^: HER2-positive; HER2: human epidermal growth factor receptor 2; HGSOC: high-grade serous ovarian carcinomas; HNC: head and neck cancers; HNSCC: head and neck squamous cell carcinoma; HNF-1α: hepatocyte nuclear factor-1α; HoFH: homozygous familial hypercholesterolemia; HPV: human papillomavirus; HSP70: heat shock protein 70; ICB: immune checkpoint blockade; ICIs: immune checkpoint inhibitors; IFN-γ: interferon gamma; IL: interleukin; IGFBP6: insulin-like growth factor binding protein 6; JAK: janus kinase; JNK: c-Jun N-terminal kinase; Keap1: Kelch-like ECH-associated protein 1; LBP: lipopolysaccharide-binding protein; LDL: low-density lipoprotein; LDLR: low-density lipoprotein receptor; Lp(a): lipoprotein(a); LPL: lipoprotein lipase; LTP: lipid transfer protein; LSECs: liver sinusoidal endothelial cells; MAPK: mitogen-activated protein kinase; MASLD: metabolic dysfunction-associated steatotic liver disease; mCRPC: metastatic CRPC; MDSC: myeloid-derived suppressor cells; MHC-I: major histocompatibility complex class I; MIF: macrophage migration inhibitory factor; MMP9: matrix metalloproteinase-9; MPR: major pathological response rate; mTOR: mechanistic target of rapamycin; mTORC1: mammalian target of rapamycin complex 1; MyD88: myeloid differentiation primary response protein 88; NF-κB: nuclear factor-B; NK: natural killer; Nrf2: nuclear factor erythroid 2-related factor 2; NSCLC: non-small cell lung cancer; OC: oral cancer; OPC: oropharyngeal cancer; ORR: objective response rate; OS: overall survival; PCSK7: proprotein convertase subtilisin/kexin type 7; pCR: pathological complete response rate; PCSK9: proprotein convertase subtilisin/kexin type 9; PD-1: programmed cell death protein 1; PDAC: pancreatic ductal adenocarcinoma; PD-L1: programmed cell death-ligand 1; PFS: progression-free survival; PI3K: phosphatidylinositol-3-kinase; PTEN: phosphatase and tensin homolog; RCC: renal cell carcinoma; S1P: sphingosine-1-phosphate; SCD1: stearoyl-CoA desaturase 1; SCLC: small-cell lung cancer; siRNA: small interfering RNA; SphK1: sphingosine kinase 1; SREBPs: sterol regulatory element-binding proteins; STAT: signal transducer and activator of transcription; TAMs: tumor-associated macrophages; TCGA: The Cancer Genome Atlas; TEAD4: TEA domain transcription factor 4; TGs: triglycerides; Tfh: T follicular helper; TKI: tyrosine kinase inhibitor; TLS: tertiary lymphoid structure; TME: tumor microenvironment; TNBC: triple-negative breast cancer; TNF-α: tumor necrosis factor alpha; Tregs: regulatory T cells; TRPV: transient receptor potential vanilloid; VASP: vasodilator-stimulated phosphoprotein; VEGF: vascular endothelial growth factor; VEGFR: vascular endothelial growth factor receptor; VLDL: very low-density lipoprotein; WHO: World Health Organization; Wnt: wingless-type MMTV integration site family; YB-1: Y-box binding protein 1.
